# Supplementary material for: Smooth Interpolating Curves with Local Control and Monotone Alternating Curvature
Source: Comput Graph Forum. 2022 Oct 6;41(5):25–38. doi: 10.1111/cgf.14600 (PMC9827861; doi:10.1111/cgf.14600)
Supplement: Supplementary file 1 — Supplement Material [file CGF-41-25-s001.zip › Local-Smooth-Interpolating-MonoCurvature/extern/clothoids/docs/api-matlab/program_listing_file_CurveBase.m.html]

Program Listing for File CurveBase.m — Clothoids v2.0.9

### Navigation

- index
- toc
- Clothoids »
- Program Listing for File CurveBase.m

# Program Listing for File CurveBase.m¶

↰ Return to documentation for file (`CurveBase.m`)

```
classdef CurveBase < handle
  %% MATLAB class wrapper for the underlying C++ class
  properties (SetAccess = protected, Hidden = true)
    mexName;
    objectHandle; % Handle to the underlying C++ class instance
  end

  methods
    function self = CurveBase( mexName )
      self.mexName = mexName;
    end
    % - - - - - - - - - - - - - - - - - - - - - - - - - - - - - - - - - - -
    function delete( self )
      %% Destroy the C++ class instance
      if self.objectHandle ~= 0
        feval( self.mexName, 'delete', self.objectHandle );
      end
      self.objectHandle = 0; % avoid double destruction of object
    end
    % - - - - - - - - - - - - - - - - - - - - - - - - - - - - - - - - - - -
    %> Return the `pointer` of the interbal stored c++ object
    %>
    %> **Usage**
    %>
    %> \rst
    %> .. code-block:: matlab
    %>
    %>   obj = ref.obj_handle();
    %>
    %> \endrst
    %>
    function obj = obj_handle( self )
      obj = self.objectHandle;
    end
    % - - - - - - - - - - - - - - - - - - - - - - - - - - - - - - - - - - -
    %> Make of copy of a curve object
    %>
    %> **Usage**
    %>
    %> \rst
    %> .. code-block:: matlab
    %>
    %>   ref.copy( C );
    %>
    %> \endrst
    %>
    %> where `C` id the curve object to be copied.
    %>
    function copy( self, C )
      feval( self.mexName, 'copy', self.objectHandle, C.obj_handle() );
    end
    % - - - - - - - - - - - - - - - - - - - - - - - - - - - - - - - - - - -
    %> Return the bounding box of the curve object
    %>
    %> **Usage**
    %>
    %> \rst
    %> .. code-block:: matlab
    %>
    %>   [ xmin, ymin, xmax, ymax ] = ref.bbox( C );
    %>   [ xmin, ymin, xmax, ymax ] = ref.bbox( C, offs );
    %>   [ xmin, ymin, xmax, ymax ] = ref.bbox( C, offs, 'ISO' );
    %>   [ xmin, ymin, xmax, ymax ] = ref.bbox( C, offs, 'SAE' );
    %>
    %> \endrst
    %>
    %> - xmin: x minimum coordinate of the bounding box
    %> - ymin: y minimum coordinate of the bounding box
    %> - xmax: x maximum coordinate of the bounding box
    %> - ymax: y maximum coordinate of the bounding box
    %>
    %> **Optional Arguments**
    %>
    %> - offs: offset of the curve used in the bbox computation
    %> - 'ISO'/'SAE': use ISO or SAE orientation of the normal for offset computation
    %>
    function [ xmin, ymin, xmax, ymax ] = bbox( self, varargin )
      % return the bounding box triangle of the circle arc
      [ xmin, ymin, xmax, ymax ] = ...
        feval( self.mexName, 'bbox', self.objectHandle, varargin{:} );
    end
    % - - - - - - - - - - - - - - - - - - - - - - - - - - - - - - - - - - -
    %> Translate the curve by `(tx,ty)`
    %>
    %> **Usage**
    %>
    %> \rst
    %> .. code-block:: matlab
    %>
    %>   ref.translate( tx, ty );
    %>
    %> \endrst
    %>
    function translate( self, tx, ty )
      % translate curve by vector (tx,ty)
      feval( self.mexName, 'translate', self.objectHandle, tx, ty );
    end
    % - - - - - - - - - - - - - - - - - - - - - - - - - - - - - - - - - - -
    %> Cut the curve at the curvilinear parameter `smin` up to `smax`
    %>
    %> **Usage**
    %>
    %>    ref.trim( smin, smax );
    %>
    function trim( self, smin, smax )
      % trim circle curve to the corresponding curvilinear parameters
      feval( self.mexName, 'trim', self.objectHandle, smin, smax );
    end
    % - - - - - - - - - - - - - - - - - - - - - - - - - - - - - - - - - - -
    %> Rotate the curve by angle `angle` around point `(cx, cy)`
    %>
    %> **Usage**
    %>
    %> \rst
    %> .. code-block:: matlab
    %>
    %>   ref.rotate( angle, cx, cy );
    %>
    %> \endrst
    %>
    function rotate( self, angle, cx, cy )
      % rotate curve around `(cx,cy)` by angle `angle`
      feval( self.mexName, 'rotate', self.objectHandle, angle, cx, cy );
    end
    % - - - - - - - - - - - - - - - - - - - - - - - - - - - - - - - - - - -
    %> Reverse the direction of travel of the curve.
    %>
    %> **Usage**
    %>
    %> \rst
    %> .. code-block:: matlab
    %>
    %>   ref.reverse();
    %>
    %> \endrst
    %>
    function reverse( self )
      % reverse curve mileage
      feval( self.mexName, 'reverse', self.objectHandle );
    end
    % - - - - - - - - - - - - - - - - - - - - - - - - - - - - - - - - - - -
    %> Scale the curve by factor `sc`
    %>
    %> **Usage**
    %>
    %> \rst
    %> .. code-block:: matlab
    %>
    %>   ref.scale( sc );
    %>
    %> \endrst
    %>
    function scale( self, sc )
      % scale curve by `sc`
      feval( self.mexName, 'scale', self.objectHandle, sc );
    end
    % - - - - - - - - - - - - - - - - - - - - - - - - - - - - - - - - - - -
    %> Translate the curve in such a way the origin is at `(newX0,newY0`.
    %>
    %> **Usage**
    %>
    %> \rst
    %> .. code-block:: matlab
    %>
    %>   ref.changeOrigin( newX0, newY0 );
    %>
    %> \endrst
    %>
    function changeOrigin( self, newX0, newY0 )
      % change the origgin or the curve to `(newX0,newY0)`
      feval( self.mexName, 'changeOrigin', self.objectHandle, newX0, newY0 );
    end
    % - - - - - - - - - - - - - - - - - - - - - - - - - - - - - - - - - - -
    %> Evaluate the curve at curvilinear coordinate `s`.
    %> Argument `s` may be a vector for multiple evaluations.
    %>
    %> **Usage**
    %>
    %> \rst
    %> .. code-block:: matlab
    %>
    %>   [ x, y, theta, kappa ] = ref.evaluate( s );
    %>   [ x, y, theta, kappa ] = ref.evaluate( s, offs );
    %>   [ x, y, theta, kappa ] = ref.evaluate( s, offs, 'ISO' );
    %>   [ x, y, theta, kappa ] = ref.evaluate( s, offs, 'SAE' );
    %>
    %> \endrst
    %>
    %> **Optional Arguments**
    %>
    %> - offs: offset of the curve used in computation
    %> - 'ISO'/'SAE': use ISO or SAE orientation of the normal for offset computation
    %>
    function [ x, y, theta, kappa ] = evaluate( self, s, varargin )
      [ x, y, theta, kappa ] = feval( self.mexName, 'evaluate', ...
         self.objectHandle, s, varargin{:} ...
      );
    end
    % - - - - - - - - - - - - - - - - - - - - - - - - - - - - - - - - - - -
    %> Evaluate the curve at curvilinear coordinate `s`.
    %> Argument `s` may be a vector for multiple evaluations.
    %>
    %> **Usage**
    %>
    %> \rst
    %> .. code-block:: matlab
    %>
    %>   XY = ref.eval( s );
    %>   XY = ref.eval( s, offs );
    %>   XY = ref.eval( s, offs, 'ISO' );
    %>   XY = ref.eval( s, offs, 'SAE' );
    %>
    %>   [X,Y] = ref.eval( s );
    %>   [X,Y] = ref.eval( s, offs );
    %>   [X,Y] = ref.eval( s, offs, 'ISO' );
    %>   [X,Y] = ref.eval( s, offs, 'SAE' );
    %>
    %> \endrst
    %>
    %> **Optional Arguments**
    %>
    %> - offs: offset of the curve used compiutation
    %> - 'ISO'/'SAE': use ISO or SAE orientation of the normal for offset computation
    %>
    %> **Output**
    %>
    %> - XY: matrix `2 x n` of the evaluated points
    %> - X: vector of the x-coordinates of the evaluated points
    %> - Y: vector of the y-coordinates of the evaluated points
    %>
    function varargout = eval( self, varargin )
      [ varargout{1:nargout} ] = ...
        feval( self.mexName, 'eval', self.objectHandle, varargin{:} );
    end
    % - - - - - - - - - - - - - - - - - - - - - - - - - - - - - - - - - - -
    %> Evaluate the first derivatives of the curve at curvilinear coordinate `s`.
    %> Argument `s` may be a vector for multiple evaluations.
    %>
    %> **Usage**
    %>
    %> \rst
    %> .. code-block:: matlab
    %>
    %>   XY = ref.eval_D( s );
    %>   XY = ref.eval_D( s, offs );
    %>   XY = ref.eval_D( s, offs, 'ISO' );
    %>   XY = ref.eval_D( s, offs, 'SAE' );
    %>
    %>   [X,Y] = ref.eval_D( s );
    %>   [X,Y] = ref.eval_D( s, offs );
    %>   [X,Y] = ref.eval_D( s, offs, 'ISO' );
    %>   [X,Y] = ref.eval_D( s, offs, 'SAE' );
    %>
    %> \endrst
    %>
    %> **Optional Arguments**
    %>
    %> - `offs`: offset of the curve used in computation
    %> - 'ISO'/'SAE': use ISO or SAE orientation of the normal for offset computation
    %>
    %> **Output**
    %>
    %> - `XY`: matrix `2 x n` of the evaluated points
    %> - `X`: vector of the x-coordinates of the evaluated point derivatives
    %> - `Y`: vector of the y-coordinates of the evaluated point derivatives
    %>
    function varargout = eval_D( self, varargin )
      % evaluate derivative at `s`
      % XY = eval_D(s,[offs,'ISO'/'SAE')
      % [X,Y] = eval_D(s,[offs,'ISO'/'SAE')
      [ varargout{1:nargout} ] = ...
        feval( self.mexName, 'eval_D', self.objectHandle, varargin{:} );
    end
    % - - - - - - - - - - - - - - - - - - - - - - - - - - - - - - - - - - -
    %> Evaluate the second derivatives of the curve at curvilinear coordinate `s`.
    %> Argument `s` may be a vector for multiple evaluations.
    %>
    %> **Usage**
    %>
    %> \rst
    %> .. code-block:: matlab
    %>
    %>   XY = ref.eval_DD( s );
    %>   XY = ref.eval_DD( s, offs );
    %>   XY = ref.eval_DD( s, offs, 'ISO' );
    %>   XY = ref.eval_DD( s, offs, 'SAE' );
    %>
    %>   [X,Y] = ref.eval_DD( s );
    %>   [X,Y] = ref.eval_DD( s, offs );
    %>   [X,Y] = ref.eval_DD( s, offs, 'ISO' );
    %>   [X,Y] = ref.eval_DD( s, offs, 'SAE' );
    %>
    %> \endrst
    %>
    %> **Optional Arguments**
    %>
    %> - `offs`: offset of the curve used in computation
    %> - 'ISO'/'SAE': use ISO or SAE orientation of the normal for offset computation
    %>
    %> **Output**
    %>
    %> - `XY`: matrix `2 x n` of the evaluated points
    %> - `X`: vector of the x-coordinates of the evaluated point derivatives
    %> - `Y`: vector of the y-coordinates of the evaluated point derivatives
    %>
    function varargout = eval_DD( self, varargin )
      [ varargout{1:nargout} ] = ...
        feval( self.mexName, 'eval_DD', self.objectHandle, varargin{:} );
    end
    % - - - - - - - - - - - - - - - - - - - - - - - - - - - - - - - - - - -
    %> Evaluate the third derivatives of the curve at curvilinear coordinate `s`.
    %> Argument `s` may be a vector for multiple evaluations.
    %>
    %> **Usage**
    %>
    %> \rst
    %> .. code-block:: matlab
    %>
    %>   XY = ref.eval_DDD( s );
    %>   XY = ref.eval_DDD( s, offs );
    %>   XY = ref.eval_DDD( s, offs, 'ISO' );
    %>   XY = ref.eval_DDD( s, offs, 'SAE' );
    %>
    %>   [X,Y] = ref.eval_DDD( s );
    %>   [X,Y] = ref.eval_DDD( s, offs );
    %>   [X,Y] = ref.eval_DDD( s, offs, 'ISO' );
    %>   [X,Y] = ref.eval_DDD( s, offs, 'SAE' );
    %>
    %> \endrst
    %>
    %> **Optional Arguments**
    %>
    %> - `offs`: offset of the curve used in computation
    %> - 'ISO'/'SAE': use ISO or SAE orientation of the normal for offset computation
    %>
    %> **Output**
    %>
    %> - `XY`: matrix `2 x n` of the evaluated points
    %> - `X`: vector of the x-coordinates of the evaluated point derivatives
    %> - `Y`: vector of the y-coordinates of the evaluated point derivatives
    %>
    function varargout = eval_DDD( self, varargin )
      [ varargout{1:nargout} ] = ...
        feval( self.mexName, 'eval_DDD', self.objectHandle, varargin{:} );
    end
    % - - - - - - - - - - - - - - - - - - - - - - - - - - - - - - - - - - -
    %> Evaluate the angle of the curve at curvilinear coordinate `s`.
    %> Argument `s` may be a vector for multiple evaluations.
    %>
    %> **Usage**
    %>
    %> \rst
    %> .. code-block:: matlab
    %>
    %>   theta = ref.theta( s );
    %>
    %> \endrst
    %>
    function th = theta(self, s)
      th = feval( self.mexName, 'theta', self.objectHandle, s );
    end
    % - - - - - - - - - - - - - - - - - - - - - - - - - - - - - - - - - - -
    %> Evaluate the angle derivatives (curvature) of the curve at curvilinear coordinate `s`.
    %> Argument `s` may be a vector for multiple evaluations.
    %>
    %> **Usage**
    %>
    %> \rst
    %> .. code-block:: matlab
    %>
    %>   theta = ref.theta_D( s );
    %>
    %> \endrst
    %>
    function th = theta_D(self, s)
      % evaluate angle derivative [curvature] at `s`
      th = feval( self.mexName, 'theta_D', self.objectHandle, s );
    end
    % - - - - - - - - - - - - - - - - - - - - - - - - - - - - - - - - - - -
    %> Evaluate the angle second derivatuve of the curve at curvilinear coordinate `s`.
    %> Argument `s` may be a vector for multiple evaluations.
    %>
    %> **Usage**
    %>
    %> \rst
    %> .. code-block:: matlab
    %>
    %>   theta = ref.theta_DD( s );
    %>
    %> \endrst
    %>
    function th = theta_DD(self, s)
      % evaluate angle second derivative at `s`
      th = feval( self.mexName, 'theta_DD', self.objectHandle, s );
    end
    % - - - - - - - - - - - - - - - - - - - - - - - - - - - - - - - - - - -
    %> Evaluate the angle third derivative of the curve at curvilinear coordinate `s`.
    %> Argument `s` may be a vector for multiple evaluations.
    %>
    %> **Usage**
    %>
    %> \rst
    %> .. code-block:: matlab
    %>
    %>   theta = ref.theta_DDD( s );
    %>
    %> \endrst
    %>
    function th = theta_DDD(self, s)
      % evaluate angle third derivative at `s`
      th = feval( self.mexName, 'theta_DDD', self.objectHandle, s );
    end
    % - - - - - - - - - - - - - - - - - - - - - - - - - - - - - - - - - - -
    %> Evaluate the curvature of the curve at curvilinear coordinate `s`.
    %> Argument `s` may be a vector for multiple evaluations.
    %>
    %> **Usage**
    %>
    %> \rst
    %> .. code-block:: matlab
    %>
    %>   theta = ref.kappa( s );
    %>
    %> \endrst
    %>
    function th = kappa(self, s)
      % evaluate curvature at `s`
      th = feval( self.mexName, 'kappa', self.objectHandle, s );
    end
    % - - - - - - - - - - - - - - - - - - - - - - - - - - - - - - - - - - -
    %> Evaluate the curvature derivative of the curve at curvilinear coordinate `s`.
    %> Argument `s` may be a vector for multiple evaluations.
    %>
    %> **Usage**
    %>
    %> \rst
    %> .. code-block:: matlab
    %>
    %>   theta = ref.kappa_D( s );
    %>
    %> \endrst
    %>
    function th = kappa_D(self, s)
      % evaluate curvature derivative at `s`
      th = feval( self.mexName, 'kappa_D', self.objectHandle, s );
    end
    % - - - - - - - - - - - - - - - - - - - - - - - - - - - - - - - - - - -
    %> Evaluate the curvature second derivative of the curve at curvilinear coordinate `s`.
    %> Argument `s` may be a vector for multiple evaluations.
    %>
    %> **Usage**
    %>
    %> \rst
    %> .. code-block:: matlab
    %>
    %>   theta = ref.kappa_DD( s );
    %>
    %> \endrst
    %>
    %>
    function th = kappa_DD(self, s)
      % evaluate curvature second derivative at `s`
      th = feval( self.mexName, 'kappa_DD', self.objectHandle, s );
    end
    % - - - - - - - - - - - - - - - - - - - - - - - - - - - - - - - - - - -
    %> Evaluate initial point of the curve.
    %>
    %> **Usage**
    %>
    %> \rst
    %> .. code-block:: matlab
    %>
    %>   [ x0, y0 ] = ref.xyBegin();
    %>
    %> \endrst
    %>
    function [ x, y ] = xyBegin( self )
      [ x, y ] = feval( self.mexName, 'xyBegin', self.objectHandle );
    end
    % - - - - - - - - - - - - - - - - - - - - - - - - - - - - - - - - - - -
    %> Evaluate final point of the curve.
    %>
    %> **Usage**
    %>
    %> \rst
    %> .. code-block:: matlab
    %>
    %>   [ x1, y1 ] = ref.xyEnd();
    %>
    %> \endrst
    %>
    function [ x, y ] = xyEnd( self )
      [ x, y ] = feval( self.mexName, 'xyEnd', self.objectHandle );
    end
    % - - - - - - - - - - - - - - - - - - - - - - - - - - - - - - - - - - -
    %> Evaluate initial x-coordinate of the curve.
    %>
    %> **Usage**
    %>
    %> \rst
    %> .. code-block:: matlab
    %>
    %>   x0 = ref.xBegin();
    %>
    %> \endrst
    %>
    function X0 = xBegin( self )
      X0 = feval( self.mexName, 'xBegin', self.objectHandle );
    end
    % - - - - - - - - - - - - - - - - - - - - - - - - - - - - - - - - - - -
    %> Evaluate final x-coordinate of the curve.
    %>
    %> **Usage**
    %>
    %> \rst
    %> .. code-block:: matlab
    %>
    %>   x1 = ref.xEnd();
    %>
    %> \endrst
    %>
    function X1 = xEnd( self )
      X1 = feval( self.mexName, 'xEnd', self.objectHandle );
    end
    % - - - - - - - - - - - - - - - - - - - - - - - - - - - - - - - - - - -
    %> Evaluate initial y-coordinate of the curve.
    %>
    %> **Usage**
    %>
    %> \rst
    %> .. code-block:: matlab
    %>
    %>   y0 = ref.yBegin();
    %>
    %> \endrst
    %>
    function Y0 = yBegin( self )
      Y0 = feval( self.mexName, 'yBegin', self.objectHandle );
    end
    % - - - - - - - - - - - - - - - - - - - - - - - - - - - - - - - - - - -
    %> Evaluate final y-coordinate of the curve.
    %>
    %> **Usage**
    %>
    %> \rst
    %> .. code-block:: matlab
    %>
    %>   y1 = ref.yEnd();
    %>
    %> \endrst
    %>
    function Y1 = yEnd( self )
      Y1 = feval( self.mexName, 'yEnd', self.objectHandle );
    end
    % - - - - - - - - - - - - - - - - - - - - - - - - - - - - - - - - - - -
    %> Evaluate initial angle of the curve.
    %>
    %> **Usage**
    %>
    %> \rst
    %> .. code-block:: matlab
    %>
    %>   theta = ref.thetaBegin();
    %>
    %> \endrst
    %>
    function th0 = thetaBegin( self )
      th0 = feval( self.mexName, 'thetaBegin', self.objectHandle );
    end
    % - - - - - - - - - - - - - - - - - - - - - - - - - - - - - - - - - - -
    %> Evaluate final angle of the curve.
    %>
    %> **Usage**
    %>
    %> \rst
    %> .. code-block:: matlab
    %>
    %>   theta = ref.thetaEnd();
    %>
    %> \endrst
    %>
    function th1 = thetaEnd( self )
      th1 = feval( self.mexName, 'thetaEnd', self.objectHandle );
    end
    % - - - - - - - - - - - - - - - - - - - - - - - - - - - - - - - - - - -
    %> Evaluate initial curvature of the curve.
    %>
    %> **Usage**
    %>
    %> \rst
    %> .. code-block:: matlab
    %>
    %>   kappa0 = ref.kappaBegin();
    %>
    %> \endrst
    %>
    function kappa0 = kappaBegin( self )
      kappa0 = feval( self.mexName, 'kappaBegin', self.objectHandle );
    end
    % - - - - - - - - - - - - - - - - - - - - - - - - - - - - - - - - - - -
    %> Evaluate final curvature of the curve.
    %>
    %> **Usage**
    %>
    %> \rst
    %> .. code-block:: matlab
    %>
    %>   kappa1 = ref.kappaEnd();
    %>
    %> \endrst
    %>
    function kappa1 = kappaEnd( self )
      kappa1 = feval( self.mexName, 'kappaEnd', self.objectHandle );
    end
    % - - - - - - - - - - - - - - - - - - - - - - - - - - - - - - - - - - -
    %> Return the length of the curve.
    %>
    %> **Usage**
    %>
    %> \rst
    %> .. code-block:: matlab
    %>
    %>   length = ref.length();
    %>
    %> \endrst
    %>
    function res = length( self, varargin )
      res = feval( self.mexName, 'length', self.objectHandle, varargin{:} );
    end
    % - - - - - - - - - - - - - - - - - - - - - - - - - - - - - - - - - - -
    function [ p1, p2 ] = points( self )
      [ p1, p2 ] = feval( self.mexName, 'points', self.objectHandle );
    end
    % - - - - - - - - - - - - - - - - - - - - - - - - - - - - - - - - - - - - -
    %> Evaluate the bounding box triangles of curve.
    %>
    %> **Usage**
    %>
    %> \rst
    %> .. code-block:: matlab
    %>
    %>   [P1,P2,P3] = ref.bbTriangles();
    %>   [P1,P2,P3] = ref.bbTriangles(max_angle,max_size);
    %>   [P1,P2,P3] = ref.bbTriangles(max_angle,max_size,offs);
    %>   [P1,P2,P3] = ref.bbTriangles(max_angle,max_size,offs,'ISO');
    %>   [P1,P2,P3] = ref.bbTriangles(max_angle,max_size,offs,'SAE');
    %>
    %> \endrst
    %>
    %> **Optional Arguments**
    %>
    %> - `max_angle`: maximum curve angle variation admitted in a triangle
    %> - `max_size`: maximum triangles size
    %> - `offs`: offset of the curve used in computation
    %> - 'ISO'/'SAE': use ISO or SAE orientation of the normal for the offset
    %>
    %> **Output**
    %>
    %> - `P1`: `2 x n` matrix with the first points of the triangles
    %> - `P2`: `2 x n` matrix with the second points of the triangles
    %> - `P3`: `2 x n` matrix with the third points of the triangles
    %>
    function [P1,P2,P3] = bbTriangles( self, varargin )
      [P1,P2,P3] = feval( self.mexName, 'bbTriangles', ...
        self.objectHandle, varargin{:} ...
      );
    end
    % - - - - - - - - - - - - - - - - - - - - - - - - - - - - - - - - - - -
    %> Evaluate the point at minimum distance of another point on the curve.
    %> `qx` and `qy` may be vectors so that the return values are vectors too.
    %>
    %> **Usage**
    %>
    %> \rst
    %> .. code-block:: matlab
    %>
    %>   [ x, y, s, t, iflag, dst ] = ref.closestPoint( qx, qy );
    %>   [ x, y, s, t, iflag, dst ] = ref.closestPoint( qx, qy, offs );
    %>   [ x, y, s, t, iflag, dst ] = ref.closestPoint( qx, qy, offs, 'ISO' );
    %>   [ x, y, s, t, iflag, dst ] = ref.closestPoint( qx, qy, offs, 'SAE' );
    %>
    %> \endrst
    %>
    %> **Optional Arguments**
    %>
    %> - offs: offset of the curve used in computation
    %> - 'ISO'/'SAE': use ISO or SAE orientation of the normal for the offset
    %>
    %> **Output**
    %>
    %> - `x`, `y`: Point at minimum distance from `(qx,qy)` on the curve.
    %> - `s`, `t`: Curvilinear coordinates of the point `(qx,qy)`.
    %> - `iflag`: `iflag < 0` some error in computation, iflag >0 is the numer of segment
    %>   containing the point at minimum distance.
    %> - `dst`: point curve distance.
    %>
    function varargout = closestPoint( self, qx, qy, varargin )
      % iflag > 0 = segment number
      [ varargout{1:nargout} ] = feval( self.mexName, 'closestPoint', ...
        self.objectHandle, qx, qy, varargin{:} ...
      );
    end
    % - - - - - - - - - - - - - - - - - - - - - - - - - - - - - - - - - - -
    %> Evaluate the distance of a point `(qx,qy)` to the curve.
    %> `qx` and `qy` may be vectors so that the return values are vectors too.
    %>
    %> **Usage**
    %>
    %> \rst
    %> .. code-block:: matlab
    %>
    %>   dst = ref.distance( qx, qy );
    %>   dst = ref.distance( qx, qy, offs );
    %>   dst = ref.distance( qx, qy, offs, 'ISO' );
    %>   dst = ref.distance( qx, qy, offs, 'SAE' );
    %>
    %> \endrst
    %>
    %> **Optional Arguments**
    %>
    %> - `offs`: offset of the curve used in computation
    %> - 'ISO'/'SAE': use ISO or SAE orientation of the normal for the offset
    %>
    function dst = distance( self, qx, qy, varargin )
      dst = feval( self.mexName, 'distance', ...
        self.objectHandle, qx, qy, varargin{:} ...
      );
    end
    % - - - - - - - - - - - - - - - - - - - - - - - - - - - - - - - - - - -
    %> Check if two curve collide.
    %>
    %> **Usage**
    %>
    %> \rst
    %> .. code-block:: matlab
    %>
    %>   ok = ref.collision( obj );
    %>   ok = ref.collision( obj, offs, offs1 );
    %>   ok = ref.collision( obj, offs, offs1, 'ISO' );
    %>   ok = ref.collision( obj, offs, offs1, 'SAE' );
    %>
    %> \endrst
    %>
    %> **Optional Arguments**
    %>
    %> - `offs`, `offs1`: offset of the curves used in computation
    %> - 'ISO'/'SAE': use ISO or SAE orientation of the normal for the offsets
    %>
    function ok = collision( self, OBJ, varargin )
      ok = feval( self.mexName, 'collision', ...
        self.objectHandle, OBJ.obj_handle(), OBJ.is_type(), varargin{:} ...
      );
    end
    % - - - - - - - - - - - - - - - - - - - - - - - - - - - - - - - - - - -
    %> Intersect two curves.
    %>
    %> **Usage**
    %>
    %> \rst
    %> .. code-block:: matlab
    %>
    %>   [s1,s2] = ref.intersect( obj );
    %>   [s1,s2] = ref.intersect( obj, offs, offs1 );
    %>   [s1,s2] = ref.intersect( obj, offs, offs1, 'ISO' );
    %>   [s1,s2] = ref.intersect( obj, offs, offs1, 'SAE' );
    %>
    %> \endrst
    %>
    %> - `s1`: curvilinear coordinates of the intersections on the first curve
    %> - `s2`: curvilinear coordinates of the intersections on the second curve
    %>
    %> **Optional Argument**
    %>
    %> - `offs`, `offs1`: offset of the curves used in computation
    %> - 'ISO'/'SAE': use ISO or SAE orientation of the normal for the offsets
    %>
    function [s1,s2] = intersect( self, OBJ, varargin )
      [s1,s2] = feval( self.mexName, 'intersect', ...
        self.objectHandle, OBJ.obj_handle(), OBJ.is_type(), varargin{:} ...
      );
    end
    % - - - - - - - - - - - - - - - - - - - - - - - - - - - - - - - - - - -
    %> Print on the console some information on the stored curve.
    %>
    %> **Usage**
    %>
    %> \rst
    %> .. code-block:: matlab
    %>
    %>   ref.info();
    %>
    %> \endrst
    %>
    function info( self )
      feval( self.mexName, 'info', self.objectHandle );
    end
    % - - - - - - - - - - - - - - - - - - - - - - - - - - - - - - - - - - -
    %> Get the curvilinear coordinates of the point `(x,y)`
    %>
    %> **Usage:**
    %>
    %> \rst
    %> .. code-block:: matlab
    %>
    %>   [s,t] = ref.find_coord( x, y );
    %>
    %> \endrst
    %>
    %> - `s`: curvilinear coordinate along the curve
    %> - `t`: curvilinear coordinate along the normal of the curve
    %>
    function [s,t] = find_coord( self, x, y )
      [s,t] = feval( self.mexName, 'findST', self.objectHandle, x, y );
    end
    % - - - - - - - - - - - - - - - - - - - - - - - - - - - - - - - - - - -
    %>
    %> Activate the use of AABB three in intersection/collision computations
    %>
    function yesAABBtree( self )
      feval( self.mexName, 'yesAABBtree', self.objectHandle );
    end
    % - - - - - - - - - - - - - - - - - - - - - - - - - - - - - - - - - - -
    %>
    %> Deactivate the use of AABB three in intersection/collision computations
    %>
    function noAABBtree( self )
      feval( self.mexName, 'noAABBtree', self.objectHandle );
    end
    % - - - - - - - - - - - - - - - - - - - - - - - - - - - - - - - - - - -
    %> Plot a triangle BBOX
    %>
    %> **Usage:**
    %>
    %> \rst
    %> .. code-block:: matlab
    %>
    %>   ref.plotTBox( P1, P2, P3 );
    %>   ref.plotTBox( P1, P2, P3, 'Color', 'red' );
    %>
    %> \endrst
    %>
    %>
    function plotTBox( self, P1, P2, P3, varargin )
      for k=1:size(P1,2)
        pp1 = P1(:,k);
        pp2 = P2(:,k);
        pp3 = P3(:,k);
        plot( [pp1(1),pp2(1),pp3(1),pp1(1)], ...
              [pp1(2),pp2(2),pp3(2),pp1(2)], varargin{:} );
      end
    end
    % - - - - - - - - - - - - - - - - - - - - - - - - - - - - - - - - - - -
    %> Plot the bounding box of the curve
    %>
    %> **Usage:**
    %>
    %> \rst
    %> .. code-block:: matlab
    %>
    %>   ref.plotBBox();
    %>   ref.plotBBox('Color', 'red' );
    %>
    %> \endrst
    %>
    function plotBBox( self, varargin )
      if nargin > 1
        offs = varargin{1};
        [xmin,ymin,xmax,ymax] = self.bbox( offs );
      else
        [xmin,ymin,xmax,ymax] = self.bbox();
      end
      x = [ xmin, xmax, xmax, xmin, xmin ];
      y = [ ymin, ymin, ymax, ymax, ymin ];
      if nargin > 2
        plot( x, y, varargin{2:end});
      else
        plot( x, y );
      end
    end
    % - - - - - - - - - - - - - - - - - - - - - - - - - - - - - - - - - - -
    %> Plot the covering triangles of the curve
    %>
    %> **Usage:**
    %>
    %> \rst
    %> .. code-block:: matlab
    %>
    %>   ref.plotTriangles()
    %>   ref.plotTriangles('red','FaceAlpha', 0.5);
    %>
    %> \endrst
    %>
    function plotTriangles( self, varargin )
      [p1,p2,p3] = self.bbTriangles();
      for k=1:size(p1,2)
        x = [ p1(1,k), p2(1,k), p3(1,k), p1(1,k) ];
        y = [ p1(2,k), p2(2,k), p3(2,k), p1(2,k) ];
        %fill( x, y, 'red','FaceAlpha', 0.5 );
        fill( x, y, varargin{:});
      end
    end
    % - - - - - - - - - - - - - - - - - - - - - - - - - - - - - - - - - - -
  end
end
```

### Quick search

### Table of Contents

- Matlab Interface Manual
- C++ API
- MATLAB API

«
hide menu

menu
sidebar
»

### Navigation

- index
- toc
- Clothoids »
- Program Listing for File CurveBase.m

© Copyright 2021, Enrico Bertolazzi and Marco Frego.
Created using Sphinx 4.2.0.
